# [^18^F]FDG Uptake in Adipose Tissue Is Not Related to Inflammation in Type 2 Diabetes Mellitus

**DOI:** 10.1007/s11307-020-01538-0

**Published:** 2020-09-04

**Authors:** Melanie Reijrink, Stefanie A. de Boer, Ines F. Antunes, Daan S. Spoor, Hiddo J. L. Heerspink, Monique E. Lodewijk, Mirjam F. Mastik, Ronald Boellaard, Marcel J. W. Greuter, Stan Benjamens, Ronald J. H. Borra, Riemer H. J. A. Slart, Jan-Luuk Hillebrands, Douwe J. Mulder

**Affiliations:** 1 Department of Vascular Medicine, University of Groningen, University Medical Center Groningen, HP AA41, Hanzeplein 1, 9700RB Groningen, The Netherlands; 2 Department of Nuclear Medicine and Molecular Imaging, Medical Imaging Center, University of Groningen, University Medical Center Groningen, Groningen, the Netherlands; 3 Department of Clinical Pharmacy and Pharmacology, University of Groningen, University Medical Center Groningen, Groningen, the Netherlands; 4 Department of Pathology and Medical Biology, Division of Pathology, University of Groningen, University Medical Center Groningen, Groningen, the Netherlands; 5 Department of Radiology and Nuclear Medicine, Amsterdam University Medical Center-VU Medical Center, Amsterdam, the Netherlands; 6 Department of Robotics and Mechatronics Biomedical Technology and Technical Medicine (MIRA), University of Twente, Enschede, the Netherlands; 7 Department of Radiology, Medical Imaging Center, University of Groningen, University Medical Center Groningen, Groningen, the Netherlands; 8 Department of Diagnostic Radiology, Medical Imaging Centre of Southwest Finland, University of Turku, Turku University Hospital, Turku, Finland; 9 Department of Biomedical Photonic Imaging, Faculty of Science and Technology, University of Twente, Enschede, the Netherlands

**Keywords:** [^18^F]FDG, GLUT, Adipose tissue, Diabetes, Insulin resistance

## Abstract

**Purpose:**

2-deoxy-2-[^18^F]fluoro-d-glucose ([^18^F]FDG) uptake is a marker of metabolic activity and is therefore used to measure the inflammatory state of several tissues. This radionuclide marker is transported through the cell membrane via glucose transport proteins (GLUTs). The aim of this study is to investigate whether insulin resistance (IR) or inflammation plays a role in [^18^F]FDG uptake in adipose tissue (AT).

**Procedures:**

This study consisted of an *in vivo* clinical part and an *ex vivo* mechanistic part. In the clinical part, [^18^F]FDG uptake in abdominal visceral AT (VAT) and subcutaneous AT (SAT) was determined using PET/CT imaging in 44 patients with early type 2 diabetes mellitus (T2DM) (age 63 [54–66] years, HbA1c [6.3 ± 0.4 %], HOMA-IR 5.1[3.1–8.5]). Plasma levels were measured with ELISA. In the mechanistic part, AT biopsies obtained from 8 patients were *ex vivo* incubated with [^18^F]FDG followed by autoradiography. Next, a qRT-PCR analysis was performed to determine GLUT and cytokine mRNA expression levels. Immunohistochemistry was performed to determine CD68^+^ macrophage infiltration and GLUT4 protein expression in AT.

**Results:**

*In vivo* VAT [^18^F]FDG uptake in patients with T2DM was inversely correlated with HOMA-IR (*r* = − 0.32, *p* = 0.034), and positively related to adiponectin plasma levels (*r* = 0.43, *p* = 0.003). *Ex vivo* [^18^F]FDG uptake in VAT was not related to CD68^+^ macrophage infiltration, and IL-1ß and IL-6 mRNA expression levels. *Ex vivo* VAT [^18^F]FDG uptake was positively related to GLUT4 (*r* = 0.83, *p* = 0.042), inversely to GLUT3 (*r* = − 0.83, *p* = 0.042) and not related to GLUT1 mRNA expression levels.

**Conclusions:**

*In vivo* [^18^F]FDG uptake in VAT from patients with T2DM is positively correlated with adiponectin levels and inversely with IR. *Ex vivo* [^18^F]FDG uptake in AT is associated with GLUT4 expression but not with pro-inflammatory markers. The effect of IR should be taken into account when interpreting data of [^18^F]FDG uptake as a marker for AT inflammation.

**Electronic supplementary material:**

The online version of this article (10.1007/s11307-020-01538-0) contains supplementary material, which is available to authorized users.

## Introduction

Abdominal obesity is strongly associated with the development of type 2 diabetes mellitus (T2DM), cardiovascular disease (CVD) and premature mortality, and therefore represents a rapidly growing threat for public health [1, 2]. Adipose tissue (AT) does not only provide storage of lipids but also functions as an endocrine organ with adipocytes that are able to secrete pro-inflammatory (*e.g.*, interleukin (IL)-6, IL-1ß) and anti-inflammatory (*e.g.*, adiponectin) cytokines, mediators also known as adipokines [3]. Also, macrophages and other immune-cells are distributed throughout the AT, and able to produce pro-inflammatory cytokines. Inflammatory processes are an important component of the development of CVD and, therefore, pro- and anti-inflammatory adipokines play a major role in the relation of AT and CVD risk [4]. Abdominal AT can be subdivided into visceral AT (VAT) and subcutaneous AT (SAT). Adiponectin levels are decreased in subjects with high VAT volume and T2DM [5]. The production of adipokines is influenced by the degree of influx of inflammatory cells, predominantly pro-inflammatory macrophages [6]. AT macrophage infiltration plays an important role in the development of insulin resistance (IR) and, therefore, T2DM [7]. Accordingly, an increased VAT inflammatory state with exaggerated adipokine production might contribute to an increased risk for developing CVD in T2DM [8–11]. Although inflammation is suspected to play a central role in AT dysfunction, to our knowledge, no validated *in vivo* method to assess the local inflammatory state of AT is currently available.

2-deoxy-2-[^18^F]fluoro-d-glucose ([^18^F]FDG) is a radiolabeled glucose analogue that enters the cell through glucose transporter (GLUT)-mediated uptake and is clinically used to assess metabolic activity performed on PET [12, 13]. In addition, [^18^F]FDG uptake in AT has been assessed as a marker of the AT inflammatory state in previous studies [13–15] which showed that [^18^F]FDG uptake was higher in VAT compared with SAT [14, 16]. Obesity has shown to decrease VAT [^18^F]FDG but not SAT [^18^F]FDG uptake, indicating that AT expansion affects glucose metabolism in VAT [16]. In fact, the presence of IR and diabetes increased SAT glucose uptake [17, 18].

The expression level of GLUT1 and GLUT3 was shown to be positively correlated with [^18^F]FDG uptake in tumour tissue [19]. Also, GLUT1 and GLUT3 are shown to play a role in immune responses [20, 21]. GLUT1 is mainly expressed in the brain, by erythrocytes and endothelial cells, whereas GLUT3 is mainly expressed in neurons and placenta [22]. In addition, GLUT1 and GLUT3 are generally insulin independent [23, 24]. AT, however, is characterized by the expression of insulin-dependent GLUT4 [25, 26]. Since AT expresses GLUTs, it is reasonable to assume that adipocytes are able to absorb [^18^F]FDG. GLUTs are dysregulated by IR in patients with T2DM [27]. Inflammatory cells play an additional important role in IR. Therefore, we hypothesise that IR affects [^18^F]FDG uptake, by affecting GLUTs, in relation to AT inflammation. The aim of this translational study was to characterize the relation of [^18^F]FDG uptake with IR and inflammation in AT.

## Materials and Methods

### Study Design

This study consisted of 2 parts: an *in vivo* clinical part in which a single-centre cross-sectional study was performed using [^18^F]FDG-PET data from the previously conducted RELEASE trial [28, 29], and a mechanistic validation study part in which *ex vivo* [^18^F]FDG uptake in AT was associated with GLUT expression and inflammatory state. The protocols of the *in vivo* [^18^F]FDG-PET/CT scan in patients with T2DM, and the *ex vivo* imaging of [^18^F]FDG incubated VAT and SAT biopsies were both reviewed and approved by the Medical Ethical Institutional Review Board of the UMCG (METC numbers 2013-080 and 2017-581, respectively). These studies were performed in compliance with the principles of the Declaration of Helsinki.

### Informed Consent

Informed consent was obtained from all individual participants included in the study.

### Study Population

Eligibility criteria for the *in vivo* imaging study were described in detail previously [28]. In short, 44 eligible patients with early T2DM without glucose-lowering drug treatment and aged between 30 and 70 years were included. T2DM was defined according to the American Diabetes Association criteria [30]. Exclusion criteria were current glucose-lowering drug use, uncontrolled hypertension (SBP > 160 mmHg or DBP > 100 mmHg) and history of CVD defined as stable coronary artery disease or acute coronary syndrome, stroke or transient ischemic attack or peripheral arterial disease.

Clinical characteristics were presented in Table 1. In summary, 61 % was male and median age was 63 years. The participants were obese (median BMI 30 [28-36]), but glycaemic indices were relatively low for a population with T2DM. The pre-scan fasting glucose level was 7.4 ± 0.97 mmol/l (133 ± 18 mg/dl).

**Table 1.** Characteristics of both study populations

	*In vivo* PET/CT type 2 diabetes mellitus cohort (*n* = 44)	*Ex vivo *^18^F-FDG uptake cohort (*n* = 8)
Male gender (*n*)	27 (61 %)	7 (88 %)
Age (years)	63 [54–66]	75 [60–77]
Diabetes duration (years)	1 [0–4]	10 [4–19]^b^
Weight (kg)	97 ± 15	87 [77–95]
BMI (kg/m^2^)	30 [28–36]	28 [24–32]
Systolic blood pressure (mmHg)	137 [127–147]	140 [132–147]
HbA_1C_ (%)	6.3 ± 0.43	7.1 [6.5–9.6]
HOMA-IR	5.1 [3.1–8.5]	
Fasting insulin (mU/l)	15.3 [9.40–23.6]	
Total cholesterol (mmol/l)	4.8 ± 0.95	
HDL (mmol/l)	1.3 [1.1–1.5]	
LDL (mmol/l)	3.1 ± 1.0	
Triglycerides (mmol/l)	1.4 [0.91–2.0]	
Fasting glucose (mmol/l)	7.4 ± 0.97^a^	
Adiponectin (ng/ml)	7.9 [6.3–10.5]	
High sensitive C-reactive protein (mg/l)	1.2 [0.70–3.1]	
Visceral adipose tissue volume (dm^3^)	7.97 [6.55–10.58]	
Subcutaneous adipose tissue volume (dm^3^)	8.86 [5.57–13.31]	
VAT-SUV_mean_	0.62 [0.56–0.72]	
SAT-SUV_mean_	0.36 [0.32–0.39]	

Data are presented as mean ± SD (when normally distributed) or as median with [IQR] (when not normally distributed). ^a^*n* = 43, ^b^*n* = 4

*BMI* body mass index, *IR* insulin resistance, *HDL* high-density lipoprotein, *LDL* low-density lipoprotein, *VAT* visceral adipose tissue, *SAT* subcutenous adipose tissue, *SUV* standardized uptake value, *FDG* fluordeoxyglucose

To investigate which type of cells within AT might be responsible for the uptake of [^18^F]FDG based on GLUT expression, and correlation with inflammatory profile markers, AT biopsies were obtained. For this indirect validation, 8 patients undergoing intestinal laparotomy surgery (for colorectal carcinoma) were included (4 with T2DM [75 % male] and 4 without T2DM [100 % male]). The main aim of this *ex vivo* study was to evaluate the association between [^18^F]FDG uptake and GLUT- and inflammatory marker expression rather than comparing T2DM with non-T2DM. Furthermore, this group was suitable for the VAT and SAT biopsies because of the negligible extra invasive actions. Exclusion criteria were presence of inflammatory diseases and T1DM. From each patient, a biopsy of ~ 1 cm^3^ from the mesenteric AT, as part of VAT, was taken from the extracted bowel part. A ~ 1 cm^3^ SAT biopsy was obtained at the end of the operation during wound closure.

### Clinical and Laboratory Assessments

The following demographic data were evaluated: age, sex, weight, BMI and blood pressure. All blood samples for the *in vivo* imaging study were obtained in the morning after at least 8 h of overnight fasting. Plasma glucose, insulin, HbA_1C_, lipid profile and high sensitive C-reactive protein (hs-CRP) were measured with routine automated assays. Adiponectin and leptin plasma levels were determined with enzyme-linked immunosorbent assay (ELISA) kits (respectively EZHADP-61 K and EZHL-80SK, Linco Research, St Charles, Mo, USA). IR was estimated with the HOMA-IR: fasting insulin × (fasting glucose / 22.5) [31].

### *In Vivo* [^18^F]FDG-PET/CT Imaging

All [^18^F]FDG-PET/CT scans were performed on a Siemens Biograph 64 slice PET/CT scanner (Siemens Medical Systems, Knoxville, TN, USA) according to the European Association of Nuclear Medicine (EANM) procedure guidelines for [^18^F]FDG imaging [32]. Participants fasted for a minimum of 8 h, and blood glucose concentrations were ensured to be less than 11 mmol/l before 3 MBq [^18^F]FDG/kg body weight was administered intravenously. A low dose (LD)CT was performed before the PET emission for anatomic localization and attenuation correction. PET emission data were acquired from the skull to knee, 3 min per bed position, 60 min post-injection [^18^F]FDG.

### *In Vivo* Adipose Tissue Analysis

All PET/LDCT measurements were performed with MATLAB software (version R2015b the MathWorks, Inc., Natick, MA, USA). In order to analyse the entire abdomen, all slices from lumbar vertebral levels L1 to L5 were manually selected. AT (dm^3^) was initially segmented by thresholding the CT images between − 174 and − 24 Hounsfield units (HU) [14, 33]. This semi-automated method was previously described in more detail [34].

[^18^F]FDG uptake in AT was determined in PET images based on the CT volumes [34], and expressed as mean standardized uptake value VAT-SUV_mean_ and SAT-SUV_mean_ respectively.

### *Ex Vivo* [^18^F]FDG Uptake in Adipose Tissue

The AT biopsies were incubated in 0.99 [0.93–1.04] MBq/ml [^18^F]FDG for 60 min at room temperature. Different incubation concentrations of unlabeled (cold) glucose were used in order to investigate if [^18^F]FDG uptake was mediated by GLUTs. The following 3 conditions were tested: no glucose, 0.25 % glucose and 2.5 % glucose. After incubation, the AT biopsies were rinsed with phosphate-buffered saline (PBS).

In order to determine [^18^F]FDG uptake *ex vivo*, autoradiography with a GE Amersham™ Typhoon™ and gamma counter with WIZARD^2^® 2480 Automatic were performed. As assessed by autoradiography, [^18^F]FDG uptake was expressed as percentage of incubated dose per area in mm^2^ (%Inc./mm^2^), calculated with OptiQuant® version 3.00. In addition, gamma counter expressed [^18^F]FDG uptake as percentage of incubated dose per gram tissue (%Inc./g). Both uptake values were corrected for background radiation.

### Immunohistochemistry

Parts of the *ex vivo* imaged AT biopsies were formalin-fixed and embedded in paraffin to study CD68^+^ macrophage infiltration and GLUT4 expression using immunohistochemistry. Sections of 3 μm thickness were cut using a microtome (RM2245, Leica Biosystem, Germany) and mounted on glass slides.

Sections were deparaffinised in xylene and rehydrated in a graded series of ethanol and heat-induced antigen retrieval was performed by incubation overnight in Tris HCL pH 9 at 80 °C (CD68) or sub-boiled for 10 min in 10 mM sodium citrate buffer pH 6 (GLUT4). After cooling down, endogenous peroxidase activity was blocked in H_2_O_2_ 0.03 % for 30 min and sections were then incubated with Monoclonal Mouse Anti-Human CD68 (PMG1, 1:250 DAKO) for 60 min at room temperature or with anti-GLUT4 antibody (Novus, pAb no. NBP1-49533, 1:100) overnight at 4 **°**C. Incubation with primary antibody CD68 was followed by incubation for 30 min with Rabbit Anti-Mouse (RAM)-HRP (P0260, DAKO Denmark) and 30 min Goat Anti-Rabbit (GAR)-HRP (P0448, DAKO, Denmark) polyclonal antibodies in 1 % human serum/1%BSA/PBS. GLUT4 primary antibody incubation was followed by incubation for 30 min with Goat Anti-Rabbit (GAR)-HRP (P0448, DAKO, Denmark) and 30 min Rabbit Anti-Goat (RAG)-HRP (P0449, DAKO, Denmark) polyclonal antibodies in 1 % human serum/1%BSA/PBS. After incubation with the chromogen 3,3′-diaminobenzidine, haematoxylin counterstaining was performed followed by dehydration with ethanol and covered with Tissue-Tek Film (Sakura Coverslipper).

Stained sections were digitalized with a NanoZoomer S360 (Hamamatsu, Japan) slide scanner. Digitalized sections were analysed with the ImageScope software package (Aperio, Leica Biosystems Imaging, USA). The standard algorithm Positive Pixel Count 2004-08-11 version 8.100 was used for quantification of positive staining. Only strong positive pixels were counted as representation of CD68^+^ macrophages and were corrected for surface area. GLUT4 staining was analysed qualitatively.

### Quantitative Reverse Transcriptase-Polymerase Chain Reaction

To determine expression of GLUTs and cytokines IL-1ß and IL-6, real-time quantitative Reverse Transcription-Polymerase Chain Reaction (qRT-PCR) was performed. No AT biopsies of 2 of the 8 patients with intestinal laparotomy surgery were snap-frozen and, therefore, 6 of the 8 patients were included in real-time qRT-PCR analysis. AT biopsies of these 6 patients were snap-frozen in − 80 °C and lysed in QIAzol Lysis Reagent. Chloroform was added to extract RNA, followed by precipitation with 100 % ethanol and washing with Buffer RWT, DNase, Buffer RPE, 80 % ethanol and RNase-free water. cDNA was synthesized using SuperScript II and 80 ng of input RNA. Real-time qRT-PCR was performed with SYBR Green using 1 ng cDNA per sample. Expression of the following genes was determined: solute carrier family 2 member (SLC2A)1 (GLUT1), SLC2A3 (GLUT3), SLC2A4 (GLUT4), IL-1ß, IL-6 and AT housekeeping genes phosphoglycerate kinase 1 (PGK1) and peptidylprolyl isomerase A (PPIA) [35]. Within a given cDNA sample, all genes were tested in triplicate. Results were expressed relative to AT housekeeping genes PGK1 and PPIA (delta crossing point (Cp) value) and expressed as 2^-dCp^. All (intron spanning) primers were designed with Primer-BLAST and UCSC Genome Browser. Primer sequences and amplicon size were shown in Supplementary Table 1.

### Statistical Analysis

Data from all included participants were used in the analysis and missing values were not imputed. Spearman’s correlation coefficient (*r*) was calculated for bivariate correlations. Medians from not-normally distributed variables were compared with the Mann-Whitney *U* test. Related samples were compared using the Wilcoxon signed-rank test. All statistical analyses were performed with IBM Statistical Package for Social Sciences (SPSS) version 23. *P* < 0.05 was considered statistically significant.

## Results

### *In Vivo* Clinical Imaging Study

#### Adipose Tissue [^18^F]FDG Uptake

**Fig. 1.**
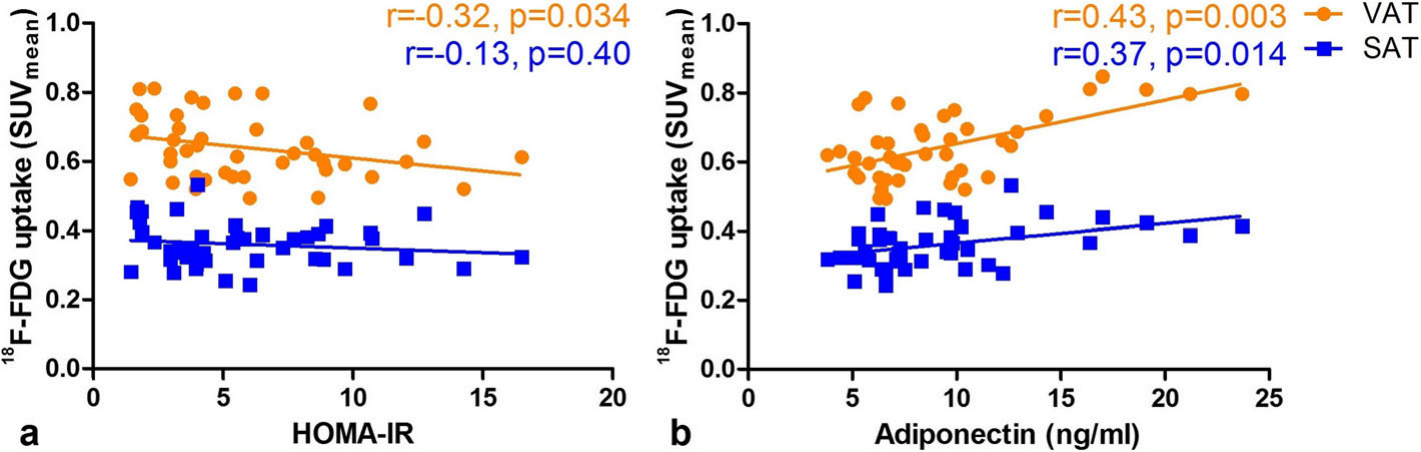
[^18^F]FDG uptake in VAT is negatively related to HOMA-IR and [^18^F]FDG uptake in adipose tissue is positively related to adiponectin. Relation between *in vivo* [^18^F]FDG uptake in mean visceral adipose tissue standardized uptake value (VAT-SUV_mean_) and mean subcutaneous adipose tissue standardized uptake value (SAT-SUV_mean_) with HOMA-insulin resistance (**a**) and plasma adiponectin levels (**b**).

In patients with T2DM, VAT-SUV_mean_ was significantly higher than SAT-SUV_mean_ (0.62 [0.56–0.72] *vs* 0.36 [0.32–0.39], *p* < 0.001). VAT-SUV_mean_ was inversely associated with HOMA-IR (*r* = − 0.32, *p* = 0.034), while SAT-SUV_mean_ was not associated with HOMA-IR (*r* = − 0.13, *p* = 0.40) (Fig. 1a). VAT-SUV_mean_ and SAT-SUV_mean_ were both significantly correlated with plasma adiponectin levels (*r* = 0.43, *p* = 0.003 and *r* = 0.37, *p* = 0.014, respectively, Fig. 1b). Adiponectin was inversely correlated with HOMA-IR (*r* = − 0.51, *p* < 0.001). Both VAT- and SAT-SUV_mean_ did not correlate with pre-scan fasting glucose (*r* = 0.088, *p* = 0.569 and *p* = 0.19, *r* = 0.223, respectively), HbA_1C_ levels and plasma hs-CRP. Leptin levels were inversely related with SAT-SUV_mean_ (*r* = − 0.31, *p* = 0.041) but not with VAT-SUV_mean_.

### Association of *Ex Vivo* [^18^F]FDG Uptake with Inflammation

#### Patient Characteristics

For the *ex vivo* part of the study, AT biopsies of 8 patients undergoing intestinal laparotomy surgery (88 % male, median age 75 years [60–77]), and median BMI 28 [24-32] were included.

#### [^18^F]FDG Uptake in Adipose Tissue

**Fig. 2.**
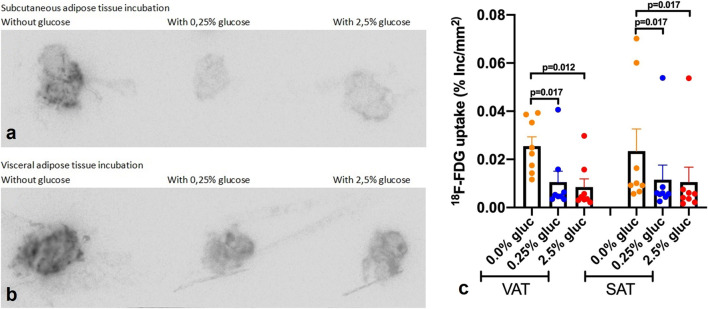
Incubation with glucose decreases [^18^F]FDG uptake in adipose tissue. Autoradiography visualized effect of *ex vivo* incubation of AT with different concentrations of glucose (0 %, 0.25 % and 2.5 %) on [^18^F]FDG uptake in subcutaneous adipose tissue (SAT) (**a**) and visceral adipose tissue (VAT) (**b**). Quantification of [^18^F]FDG uptake (in percentage of incubated dose per area in mm^2^ (%Inc./mm^2^)) in VAT and SAT, determined with autoradiography (**c**).

Since no differences in [^18^F]FDG uptake between T2DM and non-T2DM were observed, data from both groups were pooled for subsequent analyses. In contrast to clinical *in vivo* PET scan measurements, *ex vivo* VAT and SAT [^18^F]FDG uptake did not differ significantly when quantified using either autoradiography (*p* = 0.58) or the gamma counter (*p* = 0.26). As expected, the gamma counter results were in line with autoradiography data. Pre-incubation of AT with 0.25 % glucose demonstrated significantly lower [^18^F]FDG uptake compared with no glucose pre-incubation in both VAT (*p* = 0.012) and SAT (*p* = 0.017) based on quantification of the autoradiography data (Fig. 2). Incubation in the presence of a 10-fold higher glucose concentration (2.5 %) did not further decrease [^18^F]FDG uptake (Fig. 2).

#### Adipose Tissue Inflammation

To investigate if [^18^F]FDG uptake was related to the AT inflammatory state, several markers of inflammation were studied. CD68^+^ macrophages were found in a scattered and heterogeneous distribution throughout the AT. No crown-like structures [*i.e.* CD68^+^ macrophages surrounding apoptotic adipocytes] were found. No relation was found between CD68^+^ macrophage influx and autoradiography analysed [^18^F]FDG uptake in both VAT (*r* = − 0.048, *p* = 0.91) and SAT (*r* = 0.33, *p* = 0.42). Gamma counter results were in line with autoradiography data.

**Fig. 3.**
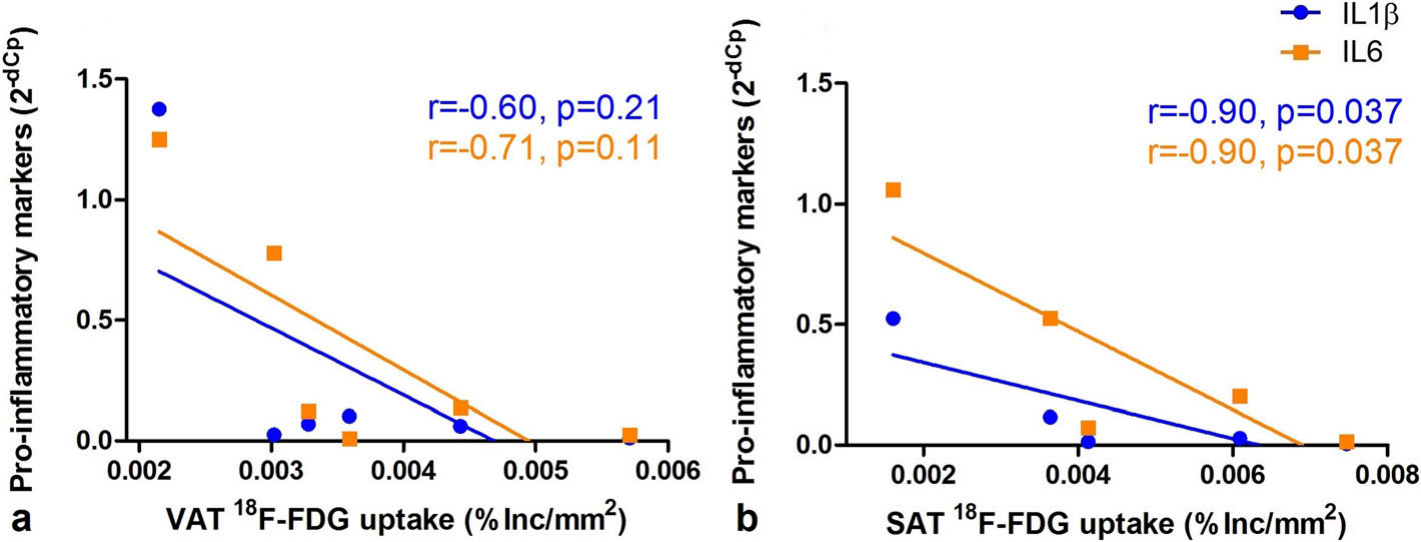
SAT [^18^F]FDG uptake is negatively related to IL expression, VAT [^18^F]FDG uptake is not related to IL expression. Association of *ex vivo* VAT (**a**) and SAT (**b**) [^18^F]FDG uptake (in percentage of incubated dose per area in mm^2^ (%Inc./mm^2^)) and with qRT-PCR determined inflammatory marker IL-1ß and IL-6 expression (expressed as 2^-dCP^).

In VAT, a trend towards an inverse relation between [^18^F]FDG uptake and IL-1ß (*r* = − 0.60, *p* = 0.21) and IL-6 (*r* = − 0.71, *p* = 0.11) mRNA expression was observed (Fig. 3a). In SAT, this inverse association between IL-1ß and IL-6 expression, and [^18^F]FDG uptake was significantly (IL-1ß (*r* = − 0.90, *p* = 0.037), IL-6 (*r* = − 0.90, *p* = 0.037) (Fig. 3b)).

Expression of the glucose transporters GLUT1 and GLUT3 in VAT was positively related to IL-6 expression (*r* = 0.81, *p* = 0.05 and *r* = 0.94, *p* = 0.005, respectively) and this pattern was also shown with IL-1ß expression levels in VAT (*r* = 0.41, *p* = 0.43 and *r* = 0.43, *p* = 0.40 respectively).

#### [^18^F]FDG Uptake and Glucose Transporter Expression

**Fig. 4.**
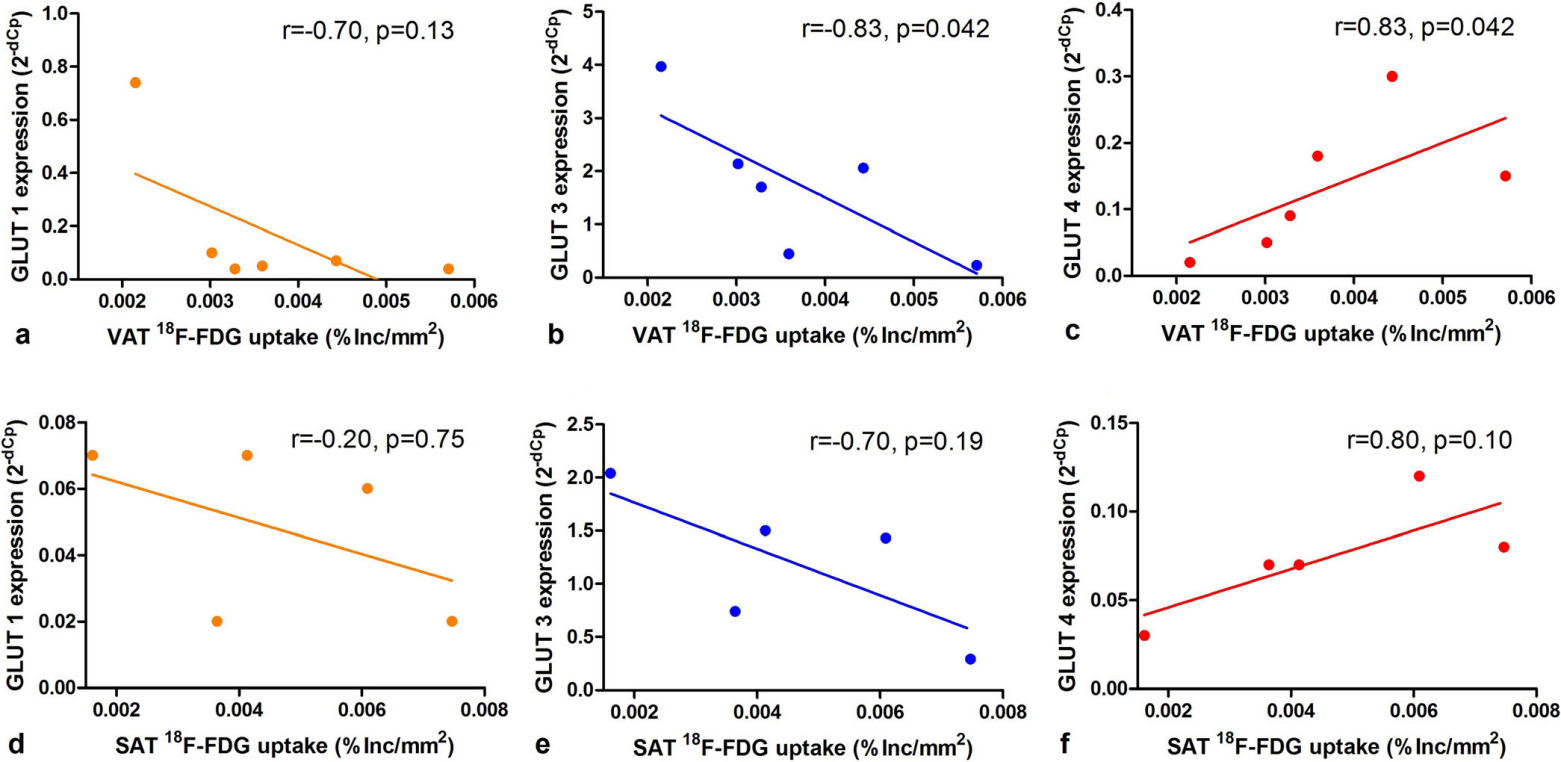
VAT [^18^F]FDG uptake is related to GLUT4 expression. Association of *ex vivo* visceral adipose tissue (VAT, panels **a**, **b**, **c**) and subcutaneous adipose tissue (SAT, panels **d**, **e**, **f**) [^18^F]FDG uptake (in percentage of incubated dose per area in mm^2^ (%Inc./mm^2^)) with mRNA expression levels of different GLUcose Transporters (GLUTs) (expressed as 2^-dCP^).

Autoradiography assessed VAT [^18^F]FDG uptake was positively related to GLUT4 expression (*r* = 0.83, *p* = 0.042, Fig. 4c), negatively to GLUT3 expression (*r* = − 0.83, *p* = 0.042, Fig. 4b) and no significant relation was found with GLUT1 expression (*r* = − 0.70, *p* = 0.13, Fig. 4a). SAT [^18^F]FDG uptake showed the same trend with respect to its relation to glucose transporter (GLUT) expression levels (GLUT1 *r* = − 0.20, *p* = 0.75, Fig. 4d; GLUT3 *r* = − 0.70, *p* = 0.19, Fig. 4e and GLUT4 *r* = 0.80, *p* = 0.10, Fig. 4f).

**Fig. 5.**
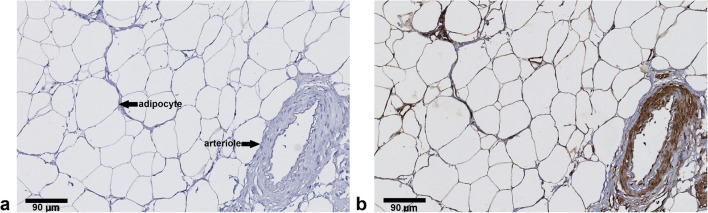
In visceral adipose tissue glucose transporter 4 (GLUT4) is expressed by adipocytes and arteriolar vascular smooth muscle cells. **a** Negative control staining (no primary anti-GLUT4 antibody added), and **b** GLUT4 staining (in brown colour) revealing cell surface expression in adipocytes and arteriolar vascular smooth muscle cells.

**Fig. 6.**
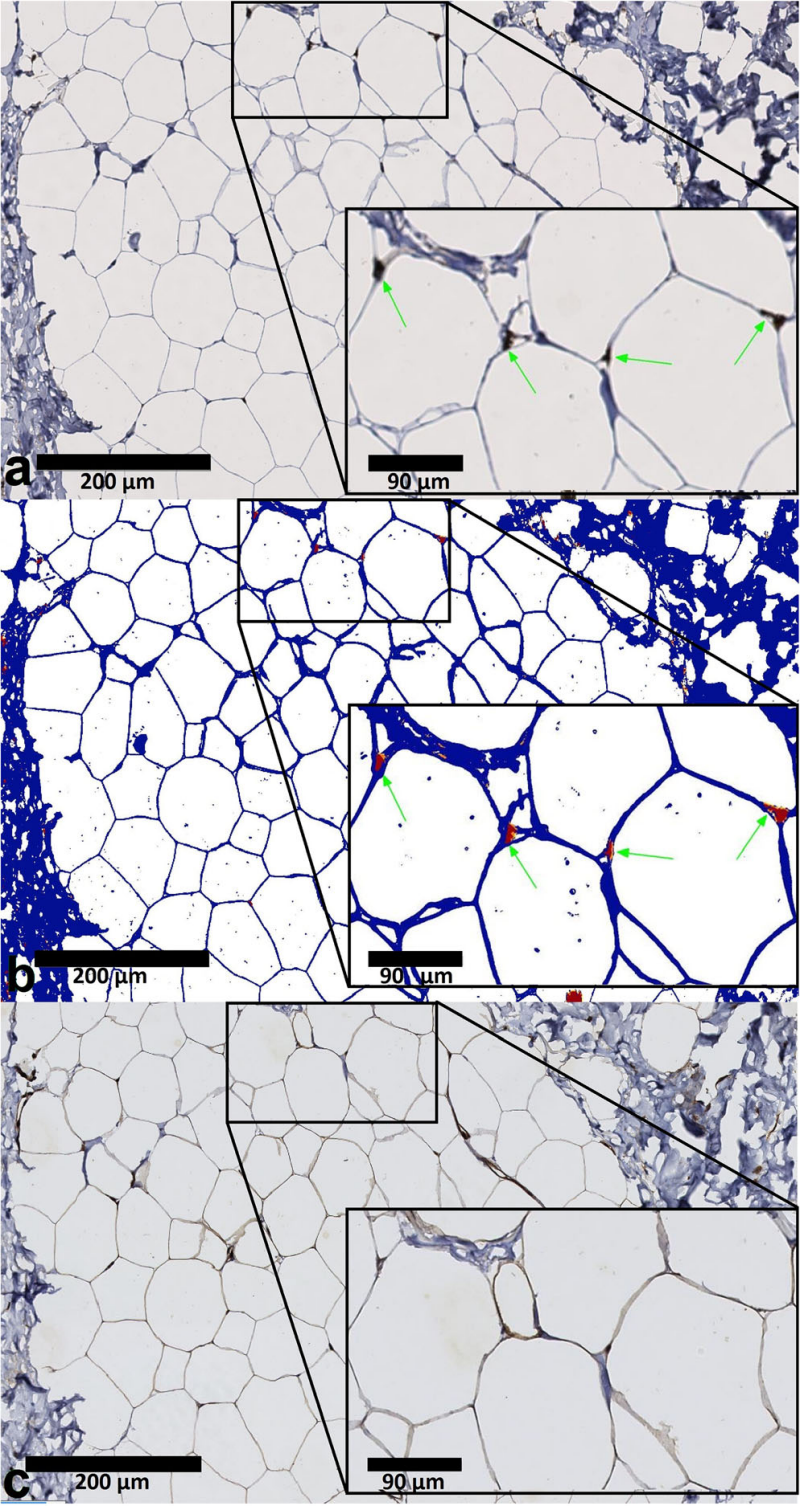
Serial sections of adipose tissue stained for CD68 and GLUT4. CD68^+^ macrophage expression with immunohistochemistry (**a**). CD68^+^ positive macrophages are brown coloured (DAB) in **a** and detected using computerized morphometry (red coloured as depicted in panel **b**, negative cells depicted in blue). Green arrows indicate CD68^+^ macrophages. An algorithm was used to quantify the amount of infiltrating macrophages. GLUT4 expression is brown coloured on the cell surface of adipocytes (**c**).

To determine spatial distribution of GLUT4 expression in AT, immunohistochemistry was performed. Cell surface GLUT4 expression was most strongly detected on adipocytes and on arteriolar medial vascular smooth muscle cells (Fig. 5 and Fig. 6).

## Discussion

With this translational study, we aimed to characterize IR and inflammation in relation to AT [^18^F]FDG uptake in patients with T2DM. *In vivo* [^18^F]FDG uptake in VAT was higher compared with SAT [^18^F]FDG uptake. Furthermore, VAT [^18^F]FDG uptake was inversely correlated with HOMA-IR, and *in vivo* VAT and SAT [^18^F]FDG uptake were positively correlated with adiponectin. *Ex vivo* SAT [^18^F]FDG uptake was inversely correlated with IL-1ß and IL-6 mRNA expression levels, and a similar trend was observed in VAT. Of the 3 GLUT’s analysed, only GLUT4 expression was positively associated with *ex vivo* [^18^F]FDG uptake. Taken together, these results indicate that VAT [^18^F]FDG uptake is insulin-dependent and not related to markers of inflammation, and therefore not a reliable marker of AT inflammation in patients with T2DM.

To our knowledge, this was the first time that [^18^F]FDG uptake in AT was investigated both *in vivo* and *ex vivo*. As expected, *in vivo* [^18^F]FDG uptake was higher in VAT compared with SAT. This finding was also reported in previous studies [16, 36]. Glucose uptake in AT was related to IR and was affected by obesity, age and gender [37]. However, it was unknown if [^18^F]FDG uptake is a surrogate marker of AT inflammation. [^18^F]FDG uptake was used as a marker of metabolic activity since cells with a high metabolic rate consume [^18^F]FDG. Low grade inflammation in AT, as noticed in patients with obesity and T2DM, was associated with increased AT metabolism [7–11]. However, in this study, we show an inverse association between inflammatory markers and *ex vivo* [^18^F]FDG uptake in AT. Our data therefore challenge the current notion that [^18^F]FDG is a marker of AT inflammation in patients with T2DM.

HOMA-IR showed to be inversely related to *in vivo* [^18^F]FDG uptake in VAT. The relation between glucose uptake in AT with obesity, insulin sensitivity and diabetes was already demonstrated before [16–18]. To substantiate these *in vivo* findings, we also performed additional *ex vivo* analyses. [^18^F]FDG uptake *ex vivo* showed to be significantly lower in AT biopsies when incubated in the presence of 0.25 % or 2.5 % glucose. Even though glucose concentrations *in vivo* were lower compared with the high concentrations in our *ex vivo* experiments, it can be suggested that glucose competes with [^18^F]FDG in the process of uptake by GLUTs.

IR was also shown to be related to the expression of several pro- and anti-inflammatory mediators. In line with previous studies [38–40], we here showed that levels of the anti-inflammatory protein adiponectin were inversely associated with IR. In addition, this study showed that adiponectin levels were positively correlated with [^18^F]FDG uptake in both VAT and SAT. An inverse association between pro-inflammatory markers with *ex vivo* SAT [^18^F]FDG uptake and no association with *ex vivo* VAT [^18^F]FDG uptake were found. Furthermore, we did not find an association between CD68^+^ macrophage infiltration and [^18^F]FDG uptake in both VAT and SAT. Based on these results, we suggest that, contrary to other tissues such as atherosclerotic plaques [41], [^18^F]FDG uptake in AT is not related to local pro-inflammatory cytokine expression and macrophage influx.

To investigate via which glucose transporter [^18^F]FDG is most likely taken up by cells residing within AT, the expression of GLUT1, GLUT3 and GLUT4 was investigated. Contrary to earlier research [19], only GLUT4, but not GLUT1 and GLUT3 mRNA expression, showed a positive association with [^18^F]FDG uptake in both VAT and SAT. Therefore, GLUT4 is most likely the most important glucose transporter for [^18^F]FDG uptake in AT. GLUT4 staining showed most intense positivity on the cell surface of adipocytes and arteriolar vascular smooth muscle cells, whereas GLUT4 expression on CD68^+^ macrophage was not unequivocally demonstrated. Therefore, it is unlikely that the positive association between increased GLUT4 mRNA expression and [^18^F]FDG uptake was due to CD68^+^ macrophage infiltration. It has been shown that macrophages express GLUT1 [21]. Our study also showed the correlation of GLUT1 and GLUT3 with inflammatory marker IL-6. However, an inverse correlation was demonstrated between GLUT3 and [^18^F]FDG uptake, and no correlation was observed between GLUT1 and [^18^F]FDG uptake. This indicates again that [^18^F]FDG uptake is at least not related to macrophage-driven AT inflammation. In order to evaluate AT macrophage infiltration, specific targeting of active macrophages, such as folate receptor-ß fluorescence imaging, or other markers which were independent of glucose metabolism are probably more accurate [42, 43]. Besides, GLUT4 is the only insulin-dependent glucose transporter in AT [26]. Increased volume of AT and increased insulin levels both result in upregulation of GLUT4 by increasing GLUT4 storage vesicles [26, 27]. However, in development of obesity, the GLUT4 recruitment to the plasma membrane reduces after a certain time [26, 44, 45]. Berger et al. showed that IR results in decreased expression of insulin-responsive glucose transporters [46]. This decreased GLUT4 expression in T2DM may also play a role in reduced [^18^F]FDG uptake in AT.

This study has some limitations. First, the study population of the *ex vivo* experiments was relatively small, and therefore, we did not assess differences between T2DM and non-T2DM. Including a larger number of subjects may result in significant findings in future studies. Despite these small groups, we did find significant associations between IL-1ß, IL-6 and GLUT4 with [^18^F]FDG uptake. Second, these participants were oncology patients and the oncogenic change can induce an inflammatory microenvironment [47]. Possibly, this effect explains the fact that we could not confirm the higher [^18^F]FDG uptake in VAT compared with SAT in the *ex vivo* experiments. Differences in tissue perfusion could also play a role *in vivo*, since SAT was located more peripherally compared with VAT [15]. Finally, IR was assessed by HOMA and not directly by the more precise glucose clamping technique.

## Conclusions

This study was designed to evaluate [^18^F]FDG uptake as a marker of AT inflammation in patients with T2DM. The current data highlight the importance of IR and its effect on GLUT4 in relation to [^18^F]FDG uptake. Furthermore, we demonstrated that [^18^F]FDG uptake was not related to CD68^+^ macrophage infiltration and inversely correlates with pro-inflammatory markers. In patients with T2DM, [^18^F]FDG uptake in AT was related to IR and this effect should be taken into account while interpreting data of [^18^F]FDG uptake as a marker for AT inflammation.

## Electronic Supplementary Material

ESM 1 (DOCX 17 kb)

## References

[1] James PT, Rigby N, Leach R, International Obesity Task Force (2004). The obesity epidemic, metabolic syndrome and future prevention strategies. Eur J Cardiovasc Prev Rehabil.

[2] Field AE, Coakley EH, Must A, Spadano JL, Laird N, Dietz WH, Rimm E, Colditz GA (2001). Impact of overweight on the risk of developing common chronic diseases during a 10-year period. Arch Intern Med.

[3] Bluher M (2013). Adipose tissue dysfunction contributes to obesity related metabolic diseases. Best Pract Res Clin Endocrinol Metab.

[4] Unamuno X, Gomez-Ambrosi J, Rodriguez A, Becerril S, Fruhbeck G, Catalan V (2018). Adipokine dysregulation and adipose tissue inflammation in human obesity. Eur J Clin Investig.

[5] Frankenberg ADV, Reis AF, Gerchman F (2017). Relationships between adiponectin levels, the metabolic syndrome, and type 2 diabetes: a literature review. Arch Endocrinol Metab.

[6] Kratz M, Coats BR, Hisert KB, Hagman D, Mutskov V, Peris E, Schoenfelt KQ, Kuzma JN, Larson I, Billing PS, Landerholm RW, Crouthamel M, Gozal D, Hwang S, Singh PK, Becker L (2014). Metabolic dysfunction drives a mechanistically distinct proinflammatory phenotype in adipose tissue macrophages. Cell Metab.

[7] Reilly SM, Saltiel AR (2017). Adapting to obesity with adipose tissue inflammation. Nat Rev Endocrinol.

[8] Kwon HW, Lee SM, Lee JW et al (2017) Association between volume and glucose metabolism of abdominal adipose tissue in healthy population. Obes Res Clin Pract (5 Suppl 1):133–143 10.1016/j.orcp.2016.12.007 28073639

[9] Liu J, Fox CS, Hickson DA (2010). Impact of abdominal visceral and subcutaneous adipose tissue on cardiometabolic risk factors: the Jackson Heart Study. J Clin Endocrinol Metab.

[10] Schnell O, Ryden L, Standl E, Ceriello A, D&CVD EASD Study Group (2016). Current perspectives on cardiovascular outcome trials in diabetes. Cardiovasc Diabetol.

[11] Hansen T, Ahlstrom H, Soderberg S (2009). Visceral adipose tissue, adiponectin levels and insulin resistance are related to atherosclerosis as assessed by whole-body magnetic resonance angiography in an elderly population. Atherosclerosis.

[12] Pimiento JM, Davis-Yadley AH, Kim RD, Chen DT, Eikman EA, Berman CG, Malafa MP (2016). Metabolic activity by 18F-FDG-PET/CT is prognostic for stage I and II pancreatic cancer. Clin Nucl Med.

[13] Bucerius J, Mani V, Wong S, Moncrieff C, Izquierdo-Garcia D, Machac J, Fuster V, Farkouh ME, Rudd JHF, Fayad ZA (2014). Arterial and fat tissue inflammation are highly correlated: a prospective 18F-FDG PET/CT study. Eur J Nucl Med Mol Imaging.

[14] Christen T, Sheikine Y, Rocha VZ, Hurwitz S, Goldfine AB, di Carli M, Libby P (2010). Increased glucose uptake in visceral versus subcutaneous adipose tissue revealed by PET imaging. JACC Cardiovasc Imaging.

[15] Virtanen KA, Lonnroth P, Parkkola R (2002). Glucose uptake and perfusion in subcutaneous and visceral adipose tissue during insulin stimulation in nonobese and obese humans. J Clin Endocrinol Metab.

[16] Oliveira AL, Azevedo DC, Bredella MA, Stanley TL, Torriani M (2015). Visceral and subcutaneous adipose tissue FDG uptake by PET/CT in metabolically healthy obese subjects. Obesity (Silver Spring).

[17] Ferrannini E, Iozzo P, Virtanen KA, Honka MJ, Bucci M, Nuutila P (2018). Adipose tissue and skeletal muscle insulin-mediated glucose uptake in insulin resistance: role of blood flow and diabetes. Am J Clin Nutr.

[18] Virtanen KA, Iozzo P, Hällsten K (2005). Increased fat mass compensates for insulin resistance in abdominal obesity and type 2 diabetes: a positron-emitting tomography study. Diabetes.

[19] Wang ZG, Yu MM, Han Y, Wu FY, Yang GJ, Li DC, Liu SM (2016). Correlation of glut-1 and glut-3 expression with F-18 FDG uptake in pulmonary inflammatory lesions. Medicine (Baltimore).

[20] Fu Y, Maianu L, Melbert BR, Garvey WT (2004). Facilitative glucose transporter gene expression in human lymphocytes, monocytes, and macrophages: a role for GLUT isoforms 1, 3, and 5 in the immune response and foam cell formation. Blood Cell Mol Dis.

[21] Freemerman AJ, Johnson AR, Sacks GN, Milner JJ, Kirk EL, Troester MA, Macintyre AN, Goraksha-Hicks P, Rathmell JC, Makowski L (2014). Metabolic reprogramming of macrophages: glucose transporter 1 (GLUT1)-mediated glucose metabolism drives a proinflammatory phenotype. J Biol Chem.

[22] McInnes M (2015). Review articles. Radiology.

[23] Simpson IA, Dwyer D, Malide D (2008). The facilitative glucose transporter GLUT3: 20 years of distinction. Am J Physiol Endocrinol Metab.

[24] Ebeling P, Koistinen HA, Koivisto VA (1998). Insulin-independent glucose transport regulates insulin sensitivity. FEBS Lett.

[25] Jaldin-Fincati JR, Pavarotti M, Frendo-Cumbo S, Bilan PJ, Klip A (2017). Update on GLUT4 vesicle traffic: a cornerstone of insulin action. Trends Endocrinol Metab.

[26] Leto D, Saltiel AR (2012). Regulation of glucose transport by insulin: traffic control of GLUT4. Nat Rev Mol Cell Biol.

[27] Correa-Giannella ML, Machado UF (2013). SLC2A4gene: a promising target for pharmacogenomics of insulin resistance. Pharmacogenomics.

[28] de Boer SA, Hovinga-de Boer MC, Heerspink HJ (2016). Arterial stiffness is positively associated with 18F-fluorodeoxyglucose positron emission tomography-assessed subclinical vascular inflammation in people with early type 2 diabetes. Diabetes Care.

[29] de Boer SA, Heerspink HJ, Juarez Orozco LE (2017). Effect of linagliptin on pulse wave velocity in early type 2 diabetes: a randomized, double-blind, controlled 26-week trial (RELEASE). Diabetes Obes Metab.

[30] Fox CS, Golden SH, Anderson C, Bray GA, Burke LE, de Boer IH, Deedwania P, Eckel RH, Ershow AG, Fradkin J, Inzucchi SE, Kosiborod M, Nelson RG, Patel MJ, Pignone M, Quinn L, Schauer PR, Selvin E, Vafiadis DK, American Heart Association Diabetes Committee of the Council on Lifestyle and Cardiometabolic Health., Council on Clinical Cardiology, Council on Cardiovascular and Stroke Nursing, Council on Cardiovascular Surgery and Anesthesia, Council on Quality of Care and Outcomes Research., American Diabetes Association (2015). Update on prevention of cardiovascular disease in adults with type 2 diabetes mellitus in light of recent evidence: a scientific statement from the American Heart Association and the American Diabetes Association. Diabetes Care.

[31] Matthews DR, Hosker JP, Rudenski AS, Naylor BA, Treacher DF, Turner RC (1985). Homeostasis model assessment: insulin resistance and beta-cell function from fasting plasma glucose and insulin concentrations in man. Diabetologia.

[32] Boellaard R, Delgado-Bolton R, Oyen WJ, Giammarile F, Tatsch K, Eschner W, Verzijlbergen FJ, Barrington SF, Pike LC, Weber WA, Stroobants S, Delbeke D, Donohoe KJ, Holbrook S, Graham MM, Testanera G, Hoekstra OS, Zijlstra J, Visser E, Hoekstra CJ, Pruim J, Willemsen A, Arends B, Kotzerke J, Bockisch A, Beyer T, Chiti A, Krause BJ, European Association of Nuclear Medicine (EANM) (2015). FDG PET/CT: EANM procedure guidelines for tumour imaging: version 2.0. Eur J Nucl Med Mol Imaging.

[33] Figueroa AL, Takx RA, MacNabb MH (2016). Relationship between measures of adiposity, arterial inflammation, and subsequent cardiovascular events. Circ Cardiovasc Imaging.

[34] de Boer SA, Spoor DS, Slart RHJA (2018). Performance evaluation of a semi-automated method for [(18)F]FDG uptake in abdominal visceral adipose tissue. Mol Imaging Biol.

[35] Neville MJ, Collins JM, Gloyn AL, McCarthy MI, Karpe F (2011). Comprehensive human adipose tissue mRNA and microRNA endogenous control selection for quantitative real-time-PCR normalization. Obesity (Silver Spring).

[36] Goncalves MD, Green-McKenzie J, Alavi A, Torigian DA (2017). Regional variation in skeletal muscle and adipose tissue FDG uptake using PET/CT and their relation to BMI. Acad Radiol.

[37] Honka MJ, Latva-Rasku A, Bucci M, Virtanen KA, Hannukainen JC, Kalliokoski KK, Nuutila P (2018). Insulin-stimulated glucose uptake in skeletal muscle, adipose tissue and liver: a positron emission tomography study. Eur J Endocrinol.

[38] Lau DC, Dhillon B, Yan H, Szmitko PE, Verma S (2005). Adipokines: molecular links between obesity and atheroslcerosis. Am J Physiol Heart Circ Physiol.

[39] Daimon M, Oizumi T, Saitoh T, Kameda W, Hirata A, Yamaguchi H, Ohnuma H, Igarashi M, Tominaga M, Kato T, Funagata study (2003). Decreased serum levels of adiponectin are a risk factor for the progression to type 2 diabetes in the Japanese Population: the Funagata study. Diabetes Care.

[40] Weyer C, Funahashi T, Tanaka S, Hotta K, Matsuzawa Y, Pratley RE, Tataranni PA (2001). Hypoadiponectinemia in obesity and type 2 diabetes: close association with insulin resistance and hyperinsulinemia. J Clin Endocrinol Metab.

[41] Masteling MG, Zeebregts CJ, Tio RA, Breek JC, Tietge UJF, de Boer JF, Glaudemans AWJM, Dierckx RAJO, Boersma HH, Slart RHJA (2011). High-resolution imaging of human atherosclerotic carotid plaques with micro 18F-FDG PET scanning exploring plaque vulnerability. J Nucl Cardiol.

[42] Jager NA, Westra J, van Dam GM, Teteloshvili N, Tio RA, Breek JC, Slart RHJA, Boersma H, Low PS, Bijl M, Zeebregts CJ (2012). Targeted folate receptor β fluorescence imaging as a measure of inflammation to estimate vulnerability within human atherosclerotic carotid plaque. J Nucl Med.

[43] Jiemy WF, Heeringa P, Kamps JAAM, van der Laken CJ, Slart RHJA, Brouwer E (2018). Positron emission tomography (PET) and single photon emission computed tomography (SPECT) imaging of macrophages in large vessel vasculitis: current status and future prospects. Autoimmun Rev.

[44] de Carvalho PP, Vargas AM, da Silva JL, Nunes MT, Machado UF (2002). GLUT4 protein is differently modulated during development of obesity in monosodium glutamate-treated mice. Life Sci.

[45] Kahn BB (1996). Lilly lecture 1995. Glucose transport: pivotal step in insulin action. Diabetes.

[46] Berger J, Biswas C, Vicario PP, Strout HV, Saperstein R, Pilch PF (1989). Decreased expression of the insulin-responsive glucose transporter in diabetes and fasting. Nature.

[47] Mantovani A, Allavena P, Sica A, Balkwill F (2008). Cancer-related inflammation. Nature.

