# Supplementary material for: [18F]FDG Uptake in Adipose Tissue Is Not Related to Inflammation in Type 2 Diabetes Mellitus
Source: Mol Imaging Biol. 2020 Sep 4;23(1):117–26. doi: 10.1007/s11307-020-01538-0 (PMC7782394; doi:10.1007/s11307-020-01538-0)
Supplement: Supplementary file 1 — (DOCX 17 kb) [file 11307_2020_1538_MOESM1_ESM.docx]

# Supplemental

Table 1: Gene data qRT-PCR.

|  | Primer Sequence forward | Primer sequence reverse | Accession number | Length amplicon (base pairs) |
| --- | --- | --- | --- | --- |
| PGK1 | GCTGGACAAGCTGGACGTTA | AGCAGCCTTAATCCTCTGGTTG | NM_000291.3 | 106 |
| PPIA | GTTCTTCGACATTGCCGTCG | AAATTTTCTGCTGTCTTTGGGACC | NM_021130.4 | 90 |
| SLC2A1 | TCACTGTCGTGTCGCTGTTT | ATGAGTATGGCACAACCCGC | NM_006516.3 | 94 |
| SLC2A3 | TTACAGCGATGGGGACACAG | GACCCCAGTGTTGTAGCCAA | NM_006931 | 98 |
| SLC2A4 | TAGGCTCCGAAGATGGGGAA | CACCTTCTGAGGGGCATTGA | NM_006516.3 | 131 |
| IL-1ß | TCGCCAGTGAAATGATGGCT | GGTCGGAGATTCGTAGCTGG | NM_000576.2 | 144 |
| IL-6 | CCACCGGGAACGAAAGAGAA | CTTGTTACATGTTTGTGGAGAAGGA | NM_001318095.1 | 79 |
